# Variation in KOOS JR improvement across total knee implant designs: a cohort study from Michigan Arthroplasty Registry Collaborative Quality Initiative

**DOI:** 10.2340/17453674.2025.44250

**Published:** 2025-08-05

**Authors:** Eric R CORNISH, Huiyong ZHENG, David C MARKEL, Brian R HALLSTROM, Richard E HUGHES

**Affiliations:** 1 MyMichigan Health, Alpena, MI; 2 Department of Orthopedic Surgery, University of Michigan, Ann Arbor, MI; 3 Henry Ford-Ascension Hospital, The Core Institute, Novi, MI, USA

## Abstract

**Background and purpose:**

Arthroplasty registries report revision risk, but patient-reported outcomes may also measure implant performance. We aimed to evaluate (i) change in patient-reported outcome measures (PROMs) across multiple total knee arthroplasty (TKA) designs in a regional registry, (ii) the association of patellar resurfacing on the change in PROMs, and (iii) the variation in PROMs change within implants with or without patellar resurfacing.

**Methods:**

This is a cohort of primary TKAs from Michigan Arthroplasty Registry Collaborative Quality Initiative (MARCQI) performed between January 1, 2017 and September 30, 2021. The dependent measure was change in KOOS JR. Independent variables were implant name and patellar resurfacing. Multivariate modeling adjusted for patient-level factors. A previous report suggests a change of 23 points in KOOS JR as clinically relevant in achieving acceptable pain/function levels. A clinically relevance ratio (CRR) of those achieving the threshold of 23 points to the overall group was calculated for each implant.

**Results:**

18 implant designs met the inclusion criteria. There were 51,606 cases with complete preoperative and postoperative KOOS JR matched pairs. There was variation in improvement from preoperative to postoperative unadjusted KOOS JR scores across implant designs (P < 0.001), ranging from 18.7 (95% confidence interval [CI] 16.8–20.6) to 27.0 (CI 24.9–29.2). Patellar resurfacing resulted in greater KOOS JR improvement 1.0 (CI 0.5–1.5, P < 0.001). Of the cases with resurfaced patellae, the CRR was 50.2% (CI 49.7–50.7). For cases without resurfaced patellae, the CRR was 47.2% (CI 45.9–48.5). The association of implant design persisted whether the patella was resurfaced or not, evident in the adjusted mean change in KOOS JR (P < 0.001), ranging from 20.1 (CI 17.6–22.6) to 25.5 (CI 24.3–26.7) for resurfaced and from 17.0 (CI 13.9–20.1) to 23.3 (CI 20.3–26.2) for not resurfaced, and the CRR difference (P < 0.001), ranging from 45.8% (CI 42.5–48.6) to 55.8% (CI 50.4–60.8) for resurfaced and from 37.9% (CI 27.4–44.7) to 51.4% (CI 43.9–56.6) for not resurfaced.

**Conclusion:**

Implant design and patellar resurfacing both show an association with KOOS JR improvement. Variations in implant design persist whether the patella is resurfaced or not. Implant selection and patellar resurfacing may be associated with patient outcomes.

Total knee arthroplasty (TKA) is a common procedure with nearly 800,000 performed annually in the USA. The American Joint Replacement Registry reported in 2022 a range of 7-year revision risk for TKA from 1.0% to 2.5% in patients aged 65 and above [1]. As a comparison, the MARCQI 7-year revision risk for TKA ranged from 1.3% to 5.4% in patients 18 years and above using data from 2012 to 2022 [2]. These results suggest that improvements may be made to reduce revision risk, but revision risk is not the only outcome patients or surgeons care about. The primary reason most patients present for TKA is pain and limitation in function. As such, patient-reported outcome measures (PROMs) have become an important part of patient care and are increasingly collected in the United States and around the world. As PROMs collection improves and the clinical use of PROMs increases, more detailed analysis of outcomes by implant and technique may be possible.

If implant design was shown to be associated with improvement in PROMs scores, it might have the potential to guide future design innovations as well as clinical decision-making in terms of implant selection and possibly whether to resurface the patella. Most studies using PROMs to compare implant designs are limited to comparing 2 implant brands [3-9], although Baker et al. [10] compared 5 knee brands. Other studies evaluate a general type of implant such as a rotating platform, anterior lip CR insert, medial stabilized, and coated implants for metal allergy.

By using data from the Michigan Arthroplasty Registry Collaborative Quality Initiative (MARCQI) the association of implant design and patellar resurfacing on PROMs was assessed. The purpose was to answer 3 questions: (i) Is TKA implant design associated with a change in PROMs, specifically KOOS JR, in a regional arthroplasty registry containing multiple implant designs? (ii) Is patellar resurfacing associated with a change in PROMs? (iii) Is there variation in the PROMs change within implant designs with or without patellar resurfacing?

## Methods

### Study design and setting

We collected data prospectively from 2012 via Michigan Arthroplasty Registry Collaborative Quality Initiative (MARCQI). MARCQI is a state-wide collaborative of surgeons, hospitals, and ambulatory surgery centers dedicated to improving the quality of care for elective hip and knee arthroplasty patients in the state of Michigan [11-13]. At the time of study, it consisted of 76 participating facilities (64 hospitals, 4 hospital outpatient departments, and 8 ambulatory surgery centers) and captured approximately 96% of arthroplasty cases throughout the state of Michigan, which has a population of 10.14 million. Our data therefore represents a reasonable proxy of the real-world outcomes of implants used by a large set of surgeons with varied practice settings and volumes in a large patient population.

The MARCQI registry contained data on 245,499 knee arthroplasty cases from February 15, 2012 to December 31, 2021. PROMs collection within MARCQI began in 2012, but MARCQI standardized collection for knees using Knee Injury and Osteoarthritis Outcome Score for Joint Replacement (KOOS JR), and Patient-Reported Outcomes Measurement Information System Global-10 (PROMIS 10) on January 1, 2015. MARCQI functions under a determination of “not regulated” status (45 CFR 46) by the University of Michigan’s Institutional Review Board as a quality improvement organization.

This study is reported according to STROBE guidelines

### Participants/study subjects

The main inclusion criteria were elective primary TKA performed between January 1, 2017 and September 10, 2021 at a participating MARCQI site (N = 115,967). Revision cases, unicompartmental surgery, or cases outside the study period (before January 1, 2017 or after September 10, 2021) were excluded. Cases needed to have preoperative and postoperative PROMs scores in the database. Implants with fewer than 500 cases in the registry, and fewer than 100 preoperative and postoperative matched pairs of KOOS JR scores were then excluded. The 100-pair threshold was based on an analysis of the data, balancing the number of implants that met the threshold and the width of the 95% confidence interval and number of degrees of freedom in the statistical models. 18 implant designs met the criteria for evaluation. The number of complete pairs and associated completion rate for each included implant design and preoperative KOOS JR scores for each implant are shown in Table 1.

**Table 1:** PROMs: pre- and postoperative pairs by implant and mean preoperative KOOS JR (standard deviation) score by implants in the study

Implant	n	Match rate	Preoperative KOOS JR mean (SD)
Persona/Persona	16,894	65	47 (13)
Triathlon/Triathlon	10,150	61	48 (13)
Triathlon/Triathlon TS	5,954	56	48 (14)
Vanguard/Maxim	4,207	63	48 (13)
Attune/Attune	3,187	63	47 (13)
Legion/Genesis II	2,631	63	48 (13)
Journey II/Journey	2,193	65	47 (13)
Evolution MP/Evolution MP	1,451	63	47 (13)
NK II GS/NK II	815	68	49 (12)
Sigma PFC/Sigma	376	50	49 (14)
LCS Complete/M.B.T.	331	65	49 (12)
NexGen LPS Option/NexGen TM	261	89	45 (13)
Journey II BCS/Journey	240	58	44 (13)
Vanguard/Maxim Mono-Lock	233	56	46 (11)
Sigma PFC/Sigma PFC	135	70	48 (11)
Sigma/M.B.T.	132	60	44 (14
iBalance/iBalance	128	48	49 (13)
NexGen Precoat/NexGen Precoat	102	47	49 (12)

### Data collected

Patient demographics (Table 2), surgical data, perioperative data, adverse event data (within 90 days of surgery), and implant catalog numbers were collected by trained clinical data abstractors at each MARCQI-specific site and uploaded to the registry database. The patients in the study group are comparable to the overall TKA population within MARCQI as evidenced by the similarity of demographic variables in both groups (Table 3). PROMs data collection is requested from all MARCQI sites, and hospitals and surgeons are incentivized by BCBSM through pay for performance and value-based reimbursement programs. The KOOS JR and PROMIS-10 PROMs were collected preoperatively and postoperatively for TKA patients. Method of collection was left to the site to determine, although a centralized digital system with email reminders was provided as an option. Sites collected PROMs using paper, tablet, and email methods. Suggested timepoints for postoperative collection were 2–16 weeks, 4–6 months, 1 year, 2 years, 4 years, out to a limit of 10 years.

**Table 2:** Descriptive statistics of variables included in the analysis

Variables	n (%)
Sex	
Female	31,732 (62)
Male	19,869 (39)
Unknown/missing	< 10 (< 0.1)
Race	
Black	3,694 (7.2)
White	46,057 (89)
Other	1,855 (3.6)
Marriage	
Yes	34,930 (68)
No	16,423 (32)
Unknown/missing	253 (0.5)
Diabetes	
Yes	10,738 (21)
No	40,843 (79)
Unknown/missing	25 (< 0.1)
Assistive devices	
Yes	13,460 (26)
No	37,802 (73)
Unknown/missing	344 (0.7)
Bleeding disorder	
Yes	427 (0.8)
No	51,169 (99)
Unknown/missing	10 (< 0.1)
History of DVT/PE	
Yes	3,888 (7.5)
No	47,704 (92)
Narcotics	
Yes	10,329 (20)
No	41,277 (80)
Unknown/missing	< 10 (< 0.1)
ASA	
I/II	24,752 (48)
III/IV	26,843 (52)
Unknown/missing	11 (< 0.1)
Primary payer	
Commercial	10,534 (20)
Medicaid	1,499 (2.9)
Medicare	28,400 (55)
Other	11,173 (22)
Education class	
Yes	37,272 (72)
No	13,895 (27)
Unknown/missing	439 (0.9)
Number of midnights	
0	4,914 (9.5)
1	29,941 (58)
≥ 2	16,751 (32)

DVT/PE = Deep vein thrombosis or pulmonary embolism; ASA = American Society of Anesthesiologists.

**Table 3:** Demographics for all primary TKA (115,967) and eligible patients for the study cohort (n = 51,606) during the study period

Variables	TKA study cohort (n = 51,606)	All primary TKA (n = 115,967)
Age ^a^	67 (9.0)	67 (9.3)
Female, n (%)	31,732 (62)	71,827 (62)
Height, cm ^a^	169 (11)	169 (11)
Weight, kg ^a^	95 (21)	95 (21)
Body mass index ^a^	33 (6.6)	33 (6.7)
Smoking status, n (%)		
Never	27,761 (54)	61,426 (53)
Previous	19,716 (38)	43,481 (38)
Current	4,035 (7.8)	10,832 (9.3)
Unknown/missing	94 (< 1)	228 (< 1)
ASA class, n (%)		
I	585 (1.1)	1,327 (1.1)
II	24,167 (47)	53,847 (46)
III	26,268 (50)	59,351 (51)
IV	575 (1.1)	1,410 (1.2)
Unknown/missing	11 (<1)	32 (< 1)
Length of stay, hours ^a^	38 (26)	
Preoperative KOOS JR ^a^	47 (13)	

a Mean (standard deviation).

ASA = American Society of Anesthesiologists.

Catalog numbers were processed using an implant library [14] provided by Curvo Labs (Evansville, IN, USA) to produce the product name for each implanted device used in the analysis. The individual catalog numbers corresponding to each implant product name are available in the supplementary materials for the 2022 MARCQI annual report [15]. A case was determined to have patellar resurfacing if a patellar component was implanted.

### Outcome

The outcome measure was the change in KOOS JR score from preoperative to postoperative. The change or delta score was the postoperative score minus the 90-day preoperative score. The postoperative scores from the date of surgery to 1 month were not used because of rapid change during this early recovery period. After 1 month, delta scores remained relatively stable with gradual changes over time. Because of the relative stability, delta scores were compared using available postoperative scores obtained anywhere between 1 month and 1 year post-surgery. If multiple postoperative scores were available, the most recent one was used, to reflect the current status of the patient.

The KOOS was developed to evaluate both the short- and long-term symptoms and function of patients with several types of knee injury and osteoarthritis. Prior to KOOS, the Lysholm knee score focused on short-term consequences and the WOMAC Osteoarthritis Index focused on long-term consequences of injury. KOOS has 42 items in 5 separately scored subscales: 1–Pain (9 items), 2–Symptoms (7 items), 3–ADL Function (17 items), 4–Sport and Recreation Function (5 items), 5–Quality of Life (4 items). It has been validated in populations with varying situations of disease, duration, age, and activity level as well as treatment including ACL reconstruction, knee arthroscopy, meniscectomy (16 years previously), and knee arthroplasty [16].

Because the KOOS survey is relatively long, it can be difficult to use in the clinic setting, often resulting in incomplete surveys and low follow-up rates. (The KOOS – PS physical function short form is shorter, but ignores pain, which is an important symptom in osteoarthritis.) To address these concerns, the Knee Injury and Osteoarthritis Outcome Score for Joint Replacement (KOOS JR) was developed as a 7-item survey to reflect knee health in measures of pain, symptom severity, and ADLs including movements or activities directly relevant and difficult with advanced knee osteoarthritis. The questions in KOOS JR are: 1 (symptom): How severe is your knee joint stiffness after first wakening in the morning?, 2 (pain): Twisting/pivoting on your knee, 3 (pain): Straightening knee fully, 4 (pain): Going up or down stairs, 5 (pain): Standing upright, 6 (ADL): Rising from sitting, 7 (ADL): Bending to floor/pick up an object. Both KOOS and KOOS JR use a 0–100 scale with 0 representing the worst and 100 the best possible knee [17].

Cowen et al. sought to interpret the composite context of KOOS JR scores, in terms of the more discrete anchor questions of the PROMIS-10 pain and function items. The least favorable “much pain, poor function” category preoperatively had mean KOOS JR scores of 40. Mixed levels of pain and function had mean scores between 46 and 55. The most favorable “less pain, good function” category had mean scores of 59. The adjusted delta to achieve the less pain level of < 3 or perform most activities was 27 (CI 22–29) on the KOOS JR scale. They suggest the lower confidence boundary of 22 as a threshold for significant clinical benefit [18].

The average improvement within the study group was 23, which approximates this threshold and was chosen for the CRR threshold. A lower threshold of 20 has been proposed [19] and adopted by CMS [20]. The lower value may reflect a sense of improvement, but not necessarily indicate adequate pain control and performance of daily activities [18].

### Statistics

Both the mean change score and clinical relevance ratio (CRR) were analyzed by implant design. The CRR dichotomizes outcomes relative to a threshold of clinical importance by reporting those that achieve the threshold at a given time point divided by the total number of patients in the study using the implant. The CRR is an attempt to minimize the effect of patients who are lost to follow-up over time [21]. Using generalized linear mixed models (GLMMs) [22,23] with logit link function for binomial distribution (achieving the improvement threshold vs not achieving the threshold in KOOS JR scores), CRR with KOOS JR scores were adjusted with models using covariates age (years old, continuous variable), sex (male, female), body mass index (BMI, less than 20, 20–30, 30–40, and ≥40), length of stay by number of midnights, race (white, black, other), marriage (yes vs no), assistive device (yes vs no), history of DVT/PE (yes vs no), narcotics (yes vs no), ASA (I/II vs III/IV), primary payer (commercial, Medicaid, Medicare, other), surgical approach (medial parapatellar, mid-vastus VMO splitting, and others), preoperative mental score on PROMIS-10 (continuous variable), preoperative physical score on PROMIS-10 (continuous variable), preoperative KOOS JR score (continuous variable), time prior to surgery for preop PROMs (weeks, continuous variable), time following surgery for postop PROMs (weeks, continuous variable). The covariates as fixed effects were determined by the fusion of clinical relevance and statistical test. Clinically, various reports note age, sex, BMI, comorbidities such as diabetes, and the ASA score to be associated with surgical risks and potential complications affecting outcomes reflected in PROMs scores. Preoperative pain and functional scores are felt to be strong predictors of postoperative pain and functional outcomes [24]. Statistically, the threshold for the covariates is P < 0.05, except for marital status (P = 0.07), preoperative KOOS JR (P = 0.5), and assistive device (P = 0.5). The implant was treated as random effect to account for the multilevel data. The 95% confidence interval (CI) for CRR was obtained by bootstrapping 100 times. The analysis was further stratified by patellar resurfacing (absent vs present). All statistical analyses were performed using SAS (Version 9.4, SAS Institute, Cary, NC, USA).

### Ethics, data sharing plan, funding, use of AI, and disclosures

MARCQI functions under a determination of “not regulated” status (45 CFR 46) by the University of Michigan’s Institutional Review Board as data is collected as part of a quality improvement organization. Patient data was anonymous within the research dataset. There was no use of AI with this study. Support for the Michigan Arthroplasty Registry Collaborative Quality Initiative is provided by Blue Cross and Blue Shield of Michigan and Blue Care Network as part of the BCBSM Value Partnerships program. This study is part of the quality improvement programs supported by BCBS, but there was no direct funding for the study. Although Blue Cross Blue Shield of Michigan and the Michigan Arthroplasty Registry Collaborative Quality Initiative work collaboratively, the opinions, beliefs, and viewpoints expressed by the author do not necessarily reflect the opinions, beliefs, and viewpoints of BCBSM or any of its employees. DM receives royalties from Smith & Nephew and is a consultant for Stryker and Smith & Nephew. Otherwise, there are no personal financial relationships that present a conflict, but salary support for 3 co-authors comes to the University of Michigan from Blue Cross Blue Shield of Michigan. Complete disclosure of interest forms according to ICMJE are available on the article page, doi: 10.2340/17453674.2025.44250

## Results

**Figure 1:**
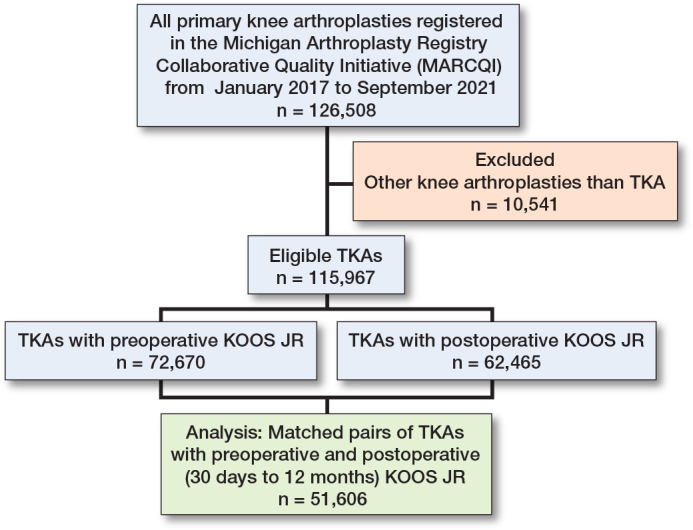
Flow diagram of cases illustrating how the final analytical dataset was generated from the MARCQI registry.

The initial cohort included 126,508 primary knee arthroplasties performed between January 2017 and September 2021. After excluding 10,541 other knee arthroplasties, 115,967 eligible TKAs remained. TKAs with preoperative KOOS JR (72,670) and TKAs with postoperative KOOS JR (62,465) were correlated, resulting in a study population of 51,606 TKAs with matched preoperative and postoperative KOOS JR (Figure 1).

### Is TKA implant design associated with a change in PROMS (KOOS JR) across implant designs?

**Figure 2:**
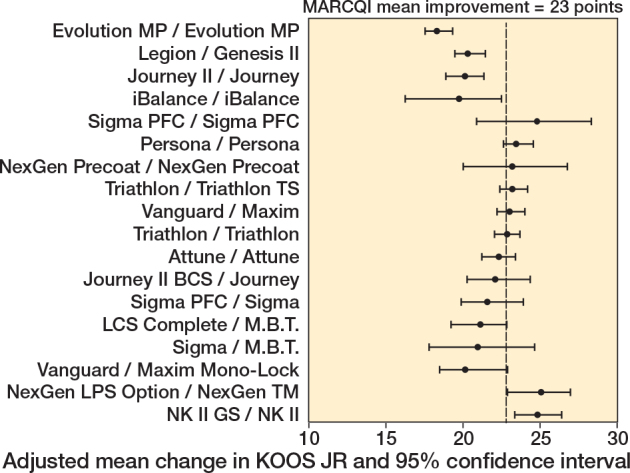
Adjusted mean change in KOOS JR. 5 implant designs had 95% confidence intervals for KOOS JR change that were below the MARCQI average of 23: Evolution MP/Evolution MP, iBalance/iBalance, Journey II/Journey, Legion/Genesis II, and Vanguard/Maxim Mono-Lock. 2 had 95% confidence intervals entirely above 23: NexGen LPS Option/NexGen TM and NK II GS/NK II. The remainder had confidence intervals that overlapped the MARCQI average.

**Figure 3:**
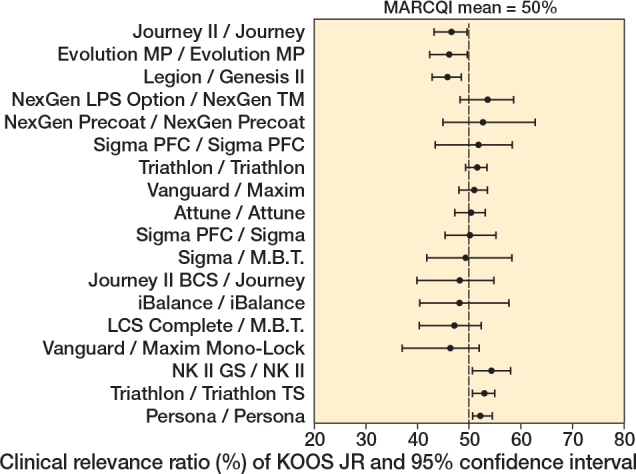
Clinical relevance ratio of KOOS JR change. 3 implant designs had 95% confidence intervals entirely below the MARCQI average CRR of 50%: Evolution MP/Evolution MP, Journey II/Journey, and Legon/Genesis II. 3 had 95% confidence intervals above the average: NK II GS/NK II, Persona/Persona, and Triathlon/Triathlon TS. The remainder had confidence intervals that overlapped the MARCQI average.

TKA implant design was associated with unadjusted KOOS JR (P < 0.001), with improvements ranging from 18.7 (CI 16.8–20.6) to 27.0 (CI 24.9–29.2), and adjusted change (P < 0.001), with improvements ranging from 20.1 (CI 17.6–22.6) to 25.5 (CI 24.3–26.7). The mean change in KOOS JR score was 23 points. Of the 18 implants, 5 had CIs for KOOS JR change that were below the MARCQI average of 23 (Figure 2). 2 had CIs entirely above 23. The remainder overlapped the MARCQI average. The CRR also showed substantial variation (Figure 3). 3 implants had CIs entirely below the MARCQI average CRR of 50%, and 3 had CIs above the average. The other 12 CIs overlapped the average.

### Is patellar resurfacing associated with a change in PROMs?

Patellar resurfacing was associated with KOOS JR improvement as well as adjusted KOOS JR improvement. The overall rate of patellar resurfacing in cases included in the MARCQI dataset that have matched preoperative and postoperative pairs was 88.6%. The mean difference in change of KOOS JR score between knees with resurfaced patellae and unresurfaced patellae in the entire MARCQI dataset was 0.87 (CI 0.39–1.34, P < 0.001), with resurfacing having the greater improvement. Only 17 implants met the requirement that the implant have at least 500 cases in the registry in which the patella was resurfaced and 100 complete KOOS JR pairs. Because of the lower frequency of unresurfaced patellae in the dataset, only 7 implants met the 500 case and 100 KOOS JR threshold. The mean difference in KOOS improvement between cases with a resurfaced patella and unresurfaced patella was 1.02 (CI 0.53–1.52, P < 0.001), with resurfacing having more improvement.

### Variation in PROMs change within implant designs with or without patellar resurfacing

**Figure 4:**
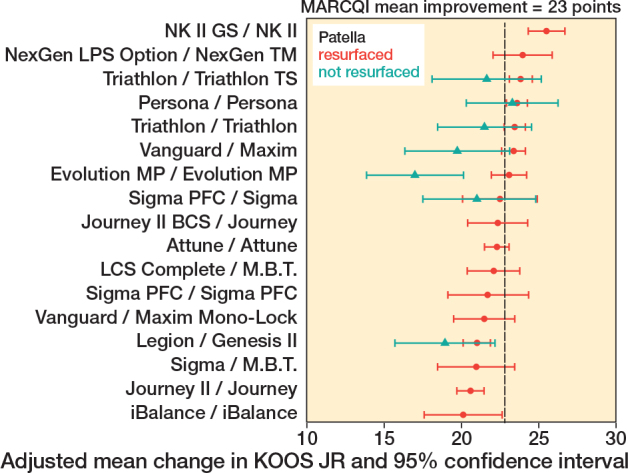
Adjusted mean change in KOOS JR in cases with patella resurfaced in red. 2 implant designs with patella resurfaced had 95% confidence intervals entirely below 23 points: Journey II/Journey and Legion/Genesis II. 6 implant designs with patella resurfaced had 95% confidence intervals entirely above the MARCQI average of 23: Triathlon/Triathlon, Triathlon/Triathlon TS, Vanguard/Maxim, Persona/Persona, NK II GS/NK II, and NexGen LPS Option/NexGen TM. The other 9 implant designs with patella resurfaced had overlapping the MARCQI average. Adjusted mean change in KOOS JR in cases with patella not resurfaced in green. 2 implant designs with patella not resurfaced had 95% confidence intervals entirely below the MARCQI average: Evolution MP/Evolution MP and Legion/Genesis II. The remaining 5 implant designs with patella not resurfaced had 95% confidence intervals crossing the MARCQI average.

**Figure 5:**
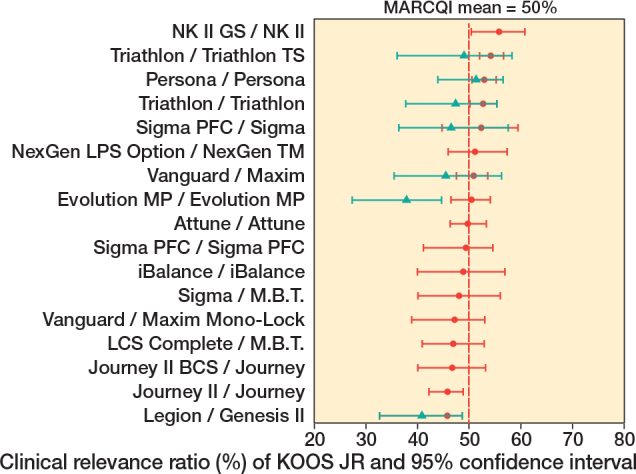
Clinical relevance ratio of KOOS JR change in cases with patella resurfaced in red. There were 2 implant designs that had CRR 95% confidence intervals entirely < 50%: Legion/Genesis II and Journey II/Journey. 2 implant designs had 95% confidence intervals entirely > 50%: Triathlon/Triathlon TS and Persona/Persona. All others had confidence intervals that overlapped 50%. Clinical relevance ratio of KOOS JR change in cases with patella not resurfaced in green. There were 2 implant designs that had CRR 95% confidence intervals entirely < 50%: Legion/Genesis II and Evolution MP/Evolution MP. All others had confidence intervals that overlapped 50%.

The association of TKA implant design with change in KOOS JR persisted within implants having the patella resurfaced (Figure 4, P < 0.001), ranging from 20.1 (CI 17.6–22.6) to 25.5 (CI 24.3–26.7) and with patella unresurfaced (Figure 4, P < 0.001) ranging from 17.0 (CI 13.9–20.1) to 23.3 (CI 20.3–26.2). CRR results also showed variation for patellar resurfacing (Figure 5), ranging from 45.8% (CI 42.5–48.6) to 55.8% (CI 50.4–60.8), and not resurfacing (Figure 5), ranging from 37.9% (CI 27.4–44.7) to 51.4% (CI 43.9–56.6). Of the cases with resurfaced patellae, the CRR was 50.2% (CI 49.7–50.7). For cases without resurfaced patellae, the CRR was 47.2% (45.9–48.5). The difference was significant at P < 0.001 level. It is noteworthy that while some implants had only a small change between patellar resurfacing and not resurfacing, the association was much more pronounced for other implants. The smallest difference in point estimates was 0.31 and the largest was 6.07 points. With patellar resurfacing, 6 implant designs had CIs entirely above the MARCQI average of 23 (Figure 4) and 2 implant designs had CIs entirely below 23 points. The other 9 implant designs overlapped the MARCQI average when the patella was resurfaced. No implant designs with patella unresurfaced had CIs entirely above MARCQI average (Figure 4). 2 implant designs with patella unresurfaced had CIs entirely below the MARCQI average. The remaining 5 implant designs, when performed without patellar resurfacing, had CIs overlapping the MARCQI average. There were 2 implants that had CRR CIs entirely < 50% with patellar resurfacing (Figure 5). There were 2 implants with CIs below 50% without patellar resurfacing (Figure 5).

## Discussion

We aimed to evaluate (i) change in patient-reported outcome measures (PROMs) across multiple total knee arthroplasty (TKA) designs in a regional registry, (ii) the association of patellar resurfacing on the change in PROMs, and (iii) the variation in PROMs change within implants with or without patellar resurfacing. We showed that there was significant variation in PROMs improvement between implant designs with 3 implants below, 3 above, and 12 overlapping the average CRR of 50% of patients achieving significant improvement. In comparison, a variety of studies investigate PROMs increases, but most focus on either a single implant [25-27] or just comparing 2 [3-9] and therefore do not give as broad an overview of the impact of implant design. Baker et al. [10] evaluated PROMs for multiple implants using data from a national arthroplasty registry, similar to this study. However, that analysis used the Oxford Knee Score and EQ-5D outcome measures, so their results are not directly comparable. That study also found variation in PROMs improvement across implant designs. The National Joint Registry’s 2022 annual report showed that implant design is associated with Oxford Knee Score, but it did not identify score change by implant brand [28].

Our findings that different brands had significant variation in PROMs improvement may be explained by manufacturer implant design features. The manufacturer’s design rationales reflect changes made in different generations to achieve better function and more normal kinematics. But different manufacturers seem to have taken different tacks: for example, the Depuy Attune offers a multi-radius transition of the distal femur and posterior condyle in contrast with the Stryker Triathlon’s single radius and shortened-flared posterior condyle to provide stability. Our results may reflect this type of variation.

Patellar resurfacing was positively associated with the change in PROMs, and although of questionable clinical significance by itself, was associated with a 3% difference in the CRR. This could affect whether a surgeon or site meets the recently set Centers for Medicare & Medicaid Services (CMS) threshold of a CRR of 60%. There are no clear changes in PROMs whether the patella is resurfaced or not [10,29,30]. Because of this, some have concluded that the tibio-femoral articulation is more important in knee outcomes [31]. However, there are indications that not resurfacing the patella may lead to anterior knee pain, higher revision rate, and complications [32]. Using AJRR data, Eiel et al. found no difference in PROMs, but an odds ratio for revision of 1.21 when not resurfacing the patella [29]. The Norwegian Arthroplasty Register reported 19% of revisions of TKAs with non-resurfaced patellae were for pain. 63% of patients with PROMs were satisfied with the result [33]. Baker et al. [10] analyzed PROMs improvement using stepwise regression modeling but did not include patellar resurfacing in the final model.

The association of TKA implant design persisted within implants having the patella resurfaced or not. The difference in adjusted mean change in KOOS JR scores and CRR was statistically significant in both cases. Variation in degree of change among implants between patellar resurfacing and not resurfacing ranged from 1.48 to 5.82.

### Limitations

We did not do a formal sensitivity analysis with multiple imputation, but our study group was comparable to the overall TKA population within MARCQI based on similar demographic variables, and a clinical relevance ratio was used to minimize the effect of patients lost to follow-up over time. Probably the most significant limitation is the small sample size for some implants. Thus, some of the confidence intervals on KOOS JR improvement are large. Nevertheless, some differences between implant designs were observed. Another related limitation was that surveys were included from 1 month to 1 year after surgery. There is a suggestion that whether the patella is resurfaced or not is associated with KOOS JR improvement. This association may be more pronounced for some implants than others. The difference noted was quite small and may not be clinically significant because of confounding by indication.

### Conclusion

Implant design and patellar resurfacing were both associated with KOOS JR improvement. Variations in implant design persist whether the patella is resurfaced or not. Implant selection and patellar resurfacing may be associated with patient outcomes. As more data accumulates, these variations could be used as early indicators in the evaluation of implant function and help direct design rationales. Clinically they may help in implant choice and potentially whether to resurface the patella.

## Notes

REH, BRH, and DCM conceived the idea of the paper. REH and ERC planned the study and drafted the initial manuscript. HZ retrieved data from MARCQI and did the statistical calculations. All authors participated in the design of the study, interpretation of the findings, improvement of the manuscript, and approved the final version.

Support for the Michigan Arthroplasty Registry Collaborative Quality Initiative is provided by Blue Cross and Blue Shield of Michigan and Blue Care Network as part of the BCBSM Value Partnerships program.

Handling co-editors: Keijo Mäkelä and Philippe Wagner

*Acta* thanks Lina Holm Ingelsrud for help with peer review of this manuscript.

## References

[1] American Joint Replacement Registry (AJRR): 2022 Annual Report. Rosemont, IL: American Academy of Orthopaedic Surgeons (AAOS); 2022. Available from: https://connect.registryapps.net/hubfs/PDFs%20and%20PPTs/2022%20AJRR%20Annual%20Report.pdf

[2] Hughes R E, Zheng H, Hallstrom B R. 2023 Michigan Arthroplasty Registry Collaborative Quality Initiative (MARCQI) Annual Report. University of Michigan, Ann Arbor. Available from: https://marcqi.org/dev/wp-content/uploads/2023/12/2023-MARCQI-ANNUAL-REPORT.pdf

[3] Lee W C, Bin Abd Razak H R, Allen J C, Chong H C, Tan H C A. Achieving minimum clinically important difference in Oxford Knee Score and Short Form-36 Physical Component Summary is less likely with single-radius compared with multiradius total knee arthroplasty in Asians. J Knee Surg 2019; 32(3): 227-32. doi: 10.1055/s-0038-1641139. 29635649

[4] Willburger R E, Oberberg S. Early and mid-term results with the ATTUNE total knee replacement system compared to PFC Sigma: a prospective comparative study. J Orthop Surg Res 2022; 17(1): 509. doi: 10.1186/s13018-022-03397-7. 36434699 PMC9694569

[5] Clarke C, Pomeroy V, Clark A, Creelman G, Hancock N, Horton S, et al. CAPAbility: comparison of the JOURNEY II Bi-Cruciate Stabilised and GENESIS II total knee arthroplasty in performance and functional ability: protocol of a randomised controlled trial. Trials 2020; 21(1): 222. doi: 10.1186/s13063-020-4143-4. 32093769 PMC7041243

[6] Rajgopal A, Aggarwal K, Kumar S. A five-year comparative functional and clinical evaluation of two contemporary cruciate-retaining knee implants. Arthroplasty Today 2020; 6(3): 369-77. doi: 10.1016/j.artd.2020.05.009. 32577480 PMC7303521

[7] Kaptein B L, den Hollander P, Thomassen B, Fiocco M, Nelissen R. A randomized controlled trial comparing tibial migration of the ATTUNE cemented cruciate-retaining knee prosthesis with the PFC-sigma design. Bone Joint J 2020; 102-B(9): 1158-66. doi: 10.1302/0301-620X.102B9.BJJ-2020-0096.R1. 32862688 PMC7468556

[8] Keiller T, Saari T, Sharegi B, KÄrrholm J. No difference in clinical outcome but in RSA in total knee arthroplasty with the Attune vs. the PFC Sigma: a randomized trial with 2-year follow-up. Acta Orthop 2023; 94: 560-9. doi: 10.2340/17453674.2023.24577. 38032279 PMC10688434

[9] Hamilton W G, Brenkel I J, Barnett S L, Allen P W, Dwyer K A, Lesko J P, et al. Comparison of existing and new total knee arthroplasty implant systems from the same manufacturer: a prospective, multicenter study. J Am Acad Orthop Surg Glob Res Rev 2021; 5(12):e21.00136. doi: 10.5435/JAAOSGlobal-D-21-00136. PMC8678005 34908561

[10] Baker P N, Deehan D J, Lees D, Jameson S, Avery P J, Gregg P J, et al. The effect of surgical factors on early patient-reported outcome measures (PROMS) following total knee replacement. J Bone Joint Surg Br 2012; 94(8): 1058-66. doi: 10.1302/0301-620X.94B8.28786. 22844046

[11] Hughes R E, Hallstrom B R, Zheng T, Kabara J, Igrisan R, Cowen M. Michigan Arthroplasty Registry Collaborative Quality Initiative (MARCQI) Report: 2012–2016. Ann Arbor: Michigan Arthroplasty Registry Collaborative Quality Initiative; 2017. Available from: https://marcqi.org/FINAL-REPORT-rev-10-28-17.pdf

[12] Hughes R E, Zheng H, Igrisan R M, Cowen M E, Markel D C, Hallstrom B R. The Michigan Arthroplasty Registry Collaborative Quality Initiative experience: improving the quality of care in Michigan. J Bone Joint Surg Am 2018; 100(22): e143. doi: 10.2106/JBJS.18.00239. 30480606

[13] Hughes R E, Hallstrom B R, Cowen M E, Igrisan R M, Singal B M, Share D A. Michigan Arthroplasty Registry Collaborative Quality Initiative (MARCQI) as a model for regional registries in the United States. Orthop Res Rev 2015; 7: 47-56. doi: 10.2147/ORR.S82732.

[14] Robertsson O, Mendenhall S, Paxton E W, Inacio M C, Graves S. Challenges in prosthesis classification. J Bone Joint Surg Am 2011; 93(Suppl 3): 72-5. doi: 10.2106/JBJS.K.00990. 22262428

[15] Hughes R E, Zheng H. 2022 MARCQI Annual Report Specifications Document. Available from: www.marcqi.org/marcqi-registry-reports-marcqi-annual-reports/

[16] Roos E M, Lohmander L S. The Knee injury and Osteoarthritis Outcome Score (KOOS): from joint injury to osteoarthritis. Health Qual Life Outcomes 2003; 1: 64. doi: 10.1186/1477-7525-1-64. 14613558 PMC280702

[17] Lyman S, Lee Y Y, Franklin P D, Wenjun L I, Cross M B, Padgett D E. Validation of the KOOS, JR: a short-form knee arthroplasty outcomes survey. Clin Orthop Relat Res 2016; 474(6): 1461-71. doi: 10.1007/s11999-016-4719-1. 26926773 PMC4868168

[18] Cowen M E, Zheng H, Hughes R E, Franklin P D, Masini M A, Hallstrom B R. How much perioperative pain and dysfunction underlie the HOOS JR and KOOS JR? Clin Orthop Relat Res 2023; 481: 1800-10. doi:10.1097/corr.0000000000002606 36917176 PMC10427044

[19] Lyman S, Lee Y Y, McLawhorn A S, Islam W, MacLean C H. What are the minimal and substantial improvements in the HOOS and KOOS and JR versions after total joint replacement? Clin Orthop Relat Res 2018; 476(12): 2432-41. doi: 10.1097/CORR.0000000000000456. 30179951 PMC6259893

[20] Ghoshal S, Harary J, Jay J F, Al-Nassir Z; BWH PROMs Workgroup; Chen A F. Evaluating patient-reported outcome measure collection and attainment of substantial clinical benefit in total joint arthroplasty patients. J Arthroplasty 2025; 40(6): 1452-9. doi: 10.1016/j.arth.2024.11.044. 39586411

[21] Orr M N, Klika A K, Gagnier J J, Bhandari M, Piuzzi N S. A call for a standardized approach to reporting patient-reported outcome measures: clinical relevance ratio. J Bone Joint Surg Am 2021; 103(22): e91. doi: 10.2106/JBJS.21.00030. 34101690

[22] Casals M, Girabent-Farrés M, Carrasco J L. Methodological quality and reporting of generalized linear mixed models in clinical medicine (2000–2012): a systematic review. PLoS One 2014; 9(11): e112653. doi: 10.1371/journal.pone.0112653. 25405342 PMC4236119

[23] Pappas M A, Spindler K P, Hu B, Higuera-Rueda C A, Rothberg M B. Volume and outcomes of joint arthroplasty. J Arthroplasty 2022; 37(11): 2128-33. doi: 10.1016/j.arth.2022.05.011. 35568138 PMC10448867

[24] Ingelsrud L H, Wilkinson J M, Overgaard S, Rolfson O, Hallstrom B, Navarro R A, et al. How do patient-reported outcome scores in international hip and knee arthroplasty registries compare? Clin Orthop Relat Res 2022; 480(10): 1884-96. doi: 10.1097/CORR.0000000000002306. 35901444 PMC9473760

[25] Mathijssen N M C, Verburg H, London N J, Landsiedl M, Dominkus M. Patient reported outcomes and implant survivorship after total knee arthroplasty with the Persona knee implant system: two year follow up. BMC Musculoskelet Disord 2019; 20(1): 97. doi: 10.1186/s12891-019-2470-y. 30832636 PMC6399845

[26] Keenan O, Brenkel I, Walmsley P. Ten-year results of the press fit condylar Sigma cobalt-chrome total knee replacement. J Knee Surg 2019; 32(3): 222-6. doi: 10.1055/s-0038-1641138. 29635648

[27] Scott C E H, Bell K R, Ng R T, MacDonald D J, Patton J T, Burnett R. Excellent 10-year patient-reported outcomes and survival in a single-radius, cruciate-retaining total knee arthroplasty. Knee Surg Sports Traumatol Arthrosc 2019; 27(4): 1106-15. doi: 10.1007/s00167-018-5179-9. 30276434 PMC6435607

[28] NJR. National Joint Registry 19th Annual Report 2022. Available from: https://www.njrcentre.org.uk/njr-annual-report-2022/36516281

[29] Eiel E S, Donnelly P, Chen A F, Sloan M. Outcomes and survivorships of total knee arthroplasty comparing resurfaced and unresurfaced patellae. J Arthroplasty 2023; 38(7 Suppl 2): S227-S232. doi: 10.1016/j.arth.2023.02.060. 36858125

[30] Robben B J, De Vries A J, Van Steenbergen L N, Nelissen R G H H, Brouwer, R W. No difference in 1-year improvement of patient-reported physical functioning and pain between resurfaced and unresurfaced patellae: analysis of 17,224 primary total knee arthroplasties in the Dutch Arthroplasty Register. Acta Orthop 2023; 94: 274-9. doi: 10.2340/17453674.2023.13430. 37291899 PMC10253942

[31] Naveen N B, Deckard E R, Ziemba-Davis M, Hanson L F, Warth L C, Meneghini R M. Patellar tilt does not affect patient reported outcomes after modern total knee arthroplasty. Knee 2022; 34: 167-77. doi: 10.1016/j.knee.2021.11.013. 34933237

[32] Grela M, Barrett M, Kunutsor S K, Blom A W, Whitehouse M R, Matharu G S. Clinical effectiveness of patellar resurfacing, no resurfacing and selective resurfacing in primary total knee replacement: systematic review and meta-analysis of interventional and observational evidence. BMC Musculoskelet Disord 2022; 23(1): 932. doi: 10.1186/s12891-022-05877-7. 36273138 PMC9587662

[33] Leta T H, Lygre S H, Skredderstuen A, Hallan G, Gjertsen J E, Rokne B, et al. Secondary patella resurfacing in painful non-resurfaced total knee arthroplasties: a study of survival and clinical outcome from the Norwegian Arthroplasty Register (1994–2011). Int Orthop 2016; 40(4): 715-22. doi: 10.1007/s00264-015-3017-y. 26493389

